# Shear stress leads to the dysfunction of endothelial cells through the Cav-1-mediated KLF2/eNOS/ERK signaling pathway under physiological conditions

**DOI:** 10.1515/biol-2022-0587

**Published:** 2023-04-15

**Authors:** Lihua Wang, Bingyue Wang, Lan Jia, Haibo Yu, Zhe Wang, Fang Wei, Aili Jiang

**Affiliations:** Department of Kidney Disease and Blood Purification Centre, 2nd Hospital of Tianjin Medical University, 23rd, Pingjiang Road, Hexi District, Tianjin 300211, China; Blood Purification Center of Tianjin Third Central Hospital, Tianjin 300170, China

**Keywords:** shear stress, dysfunction, endothelial cells, extracellular regulated protein kinase, krüppel-like factor 2, caveolin-1

## Abstract

To investigate the mechanism of shear stress on endothelial cell dysfunction for providing a theoretical basis for the reduction of arteriovenous fistula dysfunction. The *in vitro* parallel plate flow chamber was used to form different forces and shear stress to mimic the hemodynamic changes in human umbilical vein endothelial cells, and the expression and distribution of krüppel-like factor 2 (KLF2), caveolin-1 (Cav-1), p-extracellular regulated protein kinase (p-ERK), and endothelial nitric oxide synthase (eNOS) were detected by immunofluorescence and real-time quantitative polymerase chain reaction. With the prolongation of the shear stress action time, the expression of KLF2 and eNOS increased gradually, while the expression of Cav-1 and p-ERK decreased gradually. In addition, after cells were exposed to oscillatory shear stress (OSS) and low shear stress, the expression of KLF2, Cav-1, and eNOS decreased and the expression of p-ERK increased. The expression of KLF2 increased gradually with the prolongation of action time, but it was still obviously lower than that of high shear stress. Following the block of Cav-1 expression by methyl β-cyclodextrin, eNOS expression decreased, and KLF2 and p-ERK expression increased. OSS may lead to endothelial cell dysfunction by Cav-1-mediated KLF2/eNOS/ERK signaling pathway.

## 1 Introduction

Shear stress is a fundamental determinant of vascular homeostasis, regulating vascular remodeling, cardiac development, and atherogenesis [1]. Shear stress can induce endothelial cells (ECs) intracellular signaling through integrin activation to regulate vascular remodeling [2]. The fluid shear stress is also the main factor leading to the dysfunction of ECs. Vascular ECs attach to the surface of blood vessels, which first feel vascular shear stress, leading to a series of pathophysiological changes such as vascular intimal hyperplasia, lumen stenosis, and arteriovenous fistula dysfunction [3]. Different forms of shear stress can cause ECs to produce different biological effects [4]. Under the action of various shearing forces, various receptors on the ECs membrane directly sense the changes of shear force, convert mechanical signals into chemical signals, and conduct transduction through various signal pathways, transcription factor regulation, target factor action, etc., thus leading to EC dysfunction [5]. At the same time, shear stress can directly act to activate or inhibit a variety of sensitive factors such as integrin family molecules, receptor tyrosine kinases, caveolin (Cav), G protein, certain ion channels, adhesion receptors, etc. on the surface of ECs to endothelial function [6]. Sensor molecules are extremely sensitive to changes in blood flow. When the changes in intravascular shear stress are felt, they will convert these perceived mechanical signals into chemical signals, thereby activating the corresponding signal transduction and affecting the morphology and function of ECs [7–9].

Studies have confirmed that many signal pathways including mitogen-activated protein kinase (MAPK) pathway, focal adhesion kinase (FAK) pathway, protein kinase C (PKC) pathway, GTPase pathway, nuclear factor kappa-B (NF-κB) pathway, etc., act on the downstream target factors, produce a series of pathophysiological effects, realize the remodeling of ECs, and change the results and functions of ECs [10]. Other sensitive factors such as krüppel-like factor 2 (KLF2) and nuclear factor-related factor 2 (NRF2) have also been paid more and more attention [11]. Under the action of laminar shear stress, sensitive factors are activated to exert anti-inflammatory, anti-coagulant, anti-oxidant, and anti-apoptotic effects. When exposed to low shear or oscillatory shear, the expression of transcription factors is inhibited and plays a role in promoting inflammation, coagulation, oxidation, apoptosis, and proliferation, resulting in abnormal endothelial function [12]. In the present study, the mechanism of shear stress upon the ECs dysfunction was explored and the impact of shear on KLF2, Cav-1/endothelial nitric oxide synthase (eNOS)/extracellular regulated protein kinase (ERK) signaling pathways was assessed, aiming to provide a theoretical basis for the reduction of ECs dysfunction.

## 2 Materials and methods

### 2.1 Culture of human umbilical vein endothelial cells (HUVECs)

**Figure 1:**
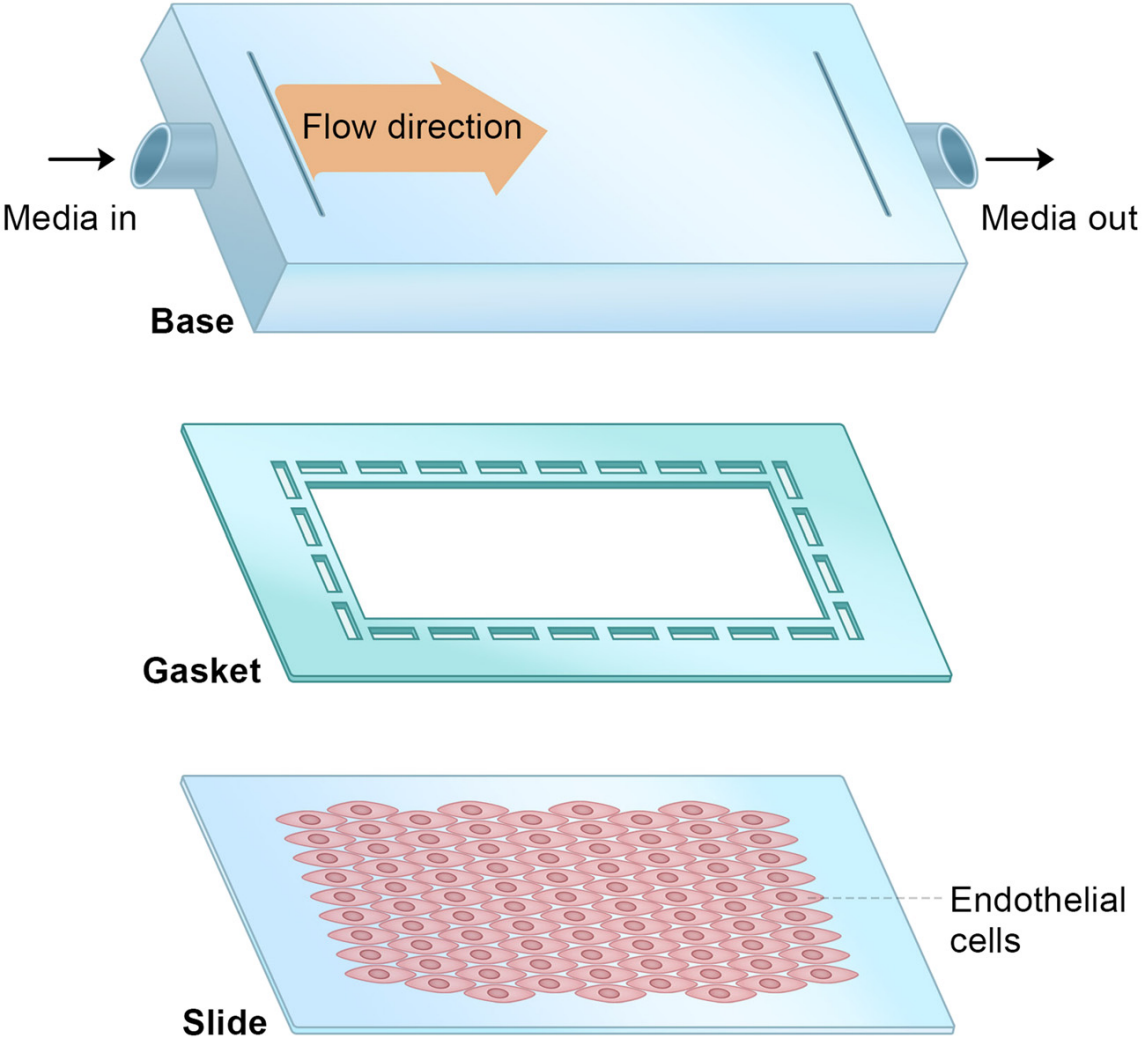
Experimental setup of EC culture.

Blood samples were collected from donors and informed consent was obtained from donors admitted to our hospital. HUVECs were isolated from blood samples of multiple donors, and cultured in Dulbecco’s modified Eagle’s medium (high sugar) with 10% fetal bovine serum and penicillin mixture under a 37°C, 5% CO_2_ cell incubator. The growth of ECs was observed every day. When ECs covered about 90% of the bottom of the culture flask, they were passed at a ratio of 1:3 in a 37°C, 5% CO_2_ incubator. Logarithmic growth phase cells were used for subsequent experiments (Figure 1).

### 2.2 Shear stress model establishment

To simulate the shear stress, a parallel plate co-culture flow chamber (Glycotech, Gaithersburg, MD, USA) was chosen, and the protocol was established as previously described [13,14]. The final density of cells was 5 × 10^6^/mL to 1 × 10^7^/mL. A constant flow pump was used to establish a laminar flow model to simulate laminar flow shear stress, and a syringe pump was utilized to establish a reciprocating flow to simulate oscillating shear stress. Two kinds of shearing forces were set with different strengths and action times, and acted on the ECs in the parallel plate flow cavity shear stress. Therefore, the experiment was divided into five groups: control, high shear stress (HSS) group, low shear stress (LSS) group, normal shear stress (NSS) group, and oscillatory shear stress (OSS) group. Control: there was no shear stress. LSS group: a constant flow pump was used to simulate laminar shear stress, and the pump flow rate was set to 18 mL/min, which simulated LSS, and the intensity was 4 dyn/cm^2^. NSS group: the pump flow rate was set to 52 mL/min, which simulated normal shearing force, and the intensity was 12 dyn/cm^2^. HSS group: the pump flow rate was set at 86 mL/min, which simulated HSS, and the intensity was 20 dyn/cm^2^. OSS group: the action of the syringe pump generated periodic fluid movement, which simulated oscillating shear stress, with an intensity of 0 ± 4 dyn/cm^2^. The calculation formula of shear stress is FSS = 6*μQ*/*wh* ^2^, where *μ* is the viscosity of the blood viscosity, *Q* (mL/min) is the volumetric flow rate through the channel, *h* is the channel height (254 μm unless otherwise noted), and *w* is the channel width (1 cm unless otherwise noted).

### 2.3 Cav-1 disruptor treatment

The ECs were treated with a Cav-1 disruptor of methyl-β-cyclodextrin (MβCD; Cat. No. 122467; Seebio Biotech, Shanghai, China) at a concentration of 20 mM for approximately 12 h.

### 2.4 Immunofluorescence detection

Cells in the 24-well plates were kept in a 37°C, 5% CO_2_ cell incubator to continue culturing, and the cell growth in the orifice plate was observed every day. After the shear stress treatment, the cells were washed with phosphate-buffered saline (PBS) at 4°C three times (each 3 min). The cells were then fixed with 4% paraformaldehyde for 15 min, and the cells were washed with PBS at 4°C for three times (each 3 min). Then cells were permeabilized with 0.25% permeabilization solution (PFA), for 15 min. After permeabilization, the supernatant was discarded, and the cells were washed at 4°C with PBS buffer three times (each 3 min). The cells were fixed again with 4% paraformaldehyde for 7 min and washed three times at 4°C with PBS buffer (each 3 min). Before the cells were incubated with the primary antibody of goat anti-rabbit IgG (Cat. No. A23420; Abbkine, CA, USA), they were blocked with serum. After blocking, the serum was discarded, and cells were incubated with primary human antibodies (anti-CAV [ab192869], anti-eNOS [ab300071], anti-p-ERK [ab201015], and anti-KLF2 [ab236507], 1:50 dilution; Abcam Technology, Cambridge, UK) and placed in a refrigerator at 4°C overnight. On the next day, the supernatant was discarded and the cells were rinsed with PBS at 4°C for three times (each 3 min), and the secondary rabbit anti-human Cav-1 monoclonal antibody (1:1,000 dilution; Cat. No. 3627S; Novusbio, CO, USA) was added and incubated in a 37°C incubator for 60 min. The supernatant was discarded, and the cells were washed with PBS at 4°C three times (each 15 min). A small drop of 4′6-diamidino-2-phenylindole at a concentration of 1 g/mL was added to the cells to stain the nucleus of the cells. The processed cells were placed under a confocal microscope (Olympus, Tokyo, Japan) to observe the antigen expression and distribution characteristics, and kept in a humidified box at 4°C.

### 2.5 Real-time quantitative polymerase chain reaction (RT-qPCR)

RT-qPCR was used to measure the mRNA expressions of KLF2, Cav-1, p-ERK, and eNOS. First, reverse transcription was carried out according to the following temperature and time program: 37°C for 30 min, 42°C for 1 h, and 72°C for 15 min. After the reaction, the cDNA was diluted with sterilized double-distilled water (ten-fold dilution) and used for subsequent qPCR analysis or stored in the refrigerator at −20°C for further studies.

Total RNA extraction was extracted by the Trizol reagent method. Briefly, after the cultured HUVECs were circulated in a parallel plate flow chamber, the culture medium was centrifuged and discarded, and the cells were washed with PBS twice. Then, 1 mL Trizol reagent was added and kept at room temperature for 5 min for complete lysis. The homogenate was transferred to a 1.5 mL Eppendorf tube, and 200 mL of chloroform was added. Then it was centrifuged at 12,000 rpm under 4°C for 15 min, and approximately 500–600 mL of supernatant was collected into a 1.5 mL Eppendorf tube. An equal volume of isopropanol was added into the supernatant, shaken evenly, and kept at room temperature for 10 min, and then centrifuged at 4°C under 12,000 rpm for 10 min. The supernatant was discarded and RNA precipitate was collected. After rinsing with 75% ethanol, the precipitate was gently collected, and centrifuged at 7,500 rpm for 5 min at 4°C. The supernatant was discarded and kept at room temperature for 10 min so that the ethanol was fully volatilized and the precipitate could be dried naturally. A portion of 20–50 μL of nuclease-free water was added to fully dissolve the precipitate and kept still for 1–2 min to obtain the RNA solution. The accumulation of fluorescent signals was used to monitor the changes in the number of amplified products in each cycle of the entire PCR amplification reaction in real-time. Finally, quantitative analysis of the starting template was conducted through the analysis of the Ct value and standard curve.

The primer sequences (GENEWIZ Technology Inc., Suzhou, China) of each gene used in this experiment are as follows: KLF2, pre-primer 5′-CTGCTCTGTCTGCCTCCAAG-3′ (Forward) and 5′-CTGCTCTCCAGGTGGGTTTC-3′ (Reverse); Cav-1, primer 5′-AACCGCGACCCTAAACACCT-3′ (Forward) and 5′-CCTTCCAAATGCCGTCAAAA-3′ (Reverse); e-NOS, 5′-ACCCTGTGCCCTGCTTCA-3′ (Forward) and 5′-GCAGGGCAAGCTGGGATCGG-3′ (Reverse); GAPDH 5′-TGTCCCCACCCCCAATGTATC-3′ (Forward) and 5′-CTCCGATGCCTGCTTCACTACCTT-3′ (Reverse).

With GAPDH as an internal reference, RT-qPCR was performed. For each pair of gene primers, triple experiments were repeated in each template. Lin Reg PCR software (Academic Medical Centre, Amsterdam, Netherlands) was employed to analyze the results and compare the expression level of each gene in different samples.

### 2.6 Statistical analysis

Data were described as the mean ± standard deviation (mean ± SD) and analyzed statistically with Statistical Product and Service Solutions (SPSS) version 19.0 (SPSS, Inc., Chicago, IL, USA). One-way analysis of variance was used to detect the differences in changes between the groups of various treatments. GraphPad Prism5 was utilized for data sorting and analysis (GraphPad Software Inc., San Diego, CA). Comparison between measurement data was conducted by *t*-test. A *p-*value less than 0.05 was considered statistically significant.


**Informed consent:** Informed consent has been obtained from all individuals included in this study.
**Ethical approval:** The research related to human use has been complied with all the relevant national regulations, institutional policies and in accordance with the tenets of the Helsinki Declaration, and has been approved by the Ethics Committee of the 2nd Hospital of Tianjin Medical University.

## 3 Results

### 3.1 The effect of shear stress on the expression of KLF2 in HUVEC under physiological conditions

**Figure 2:**
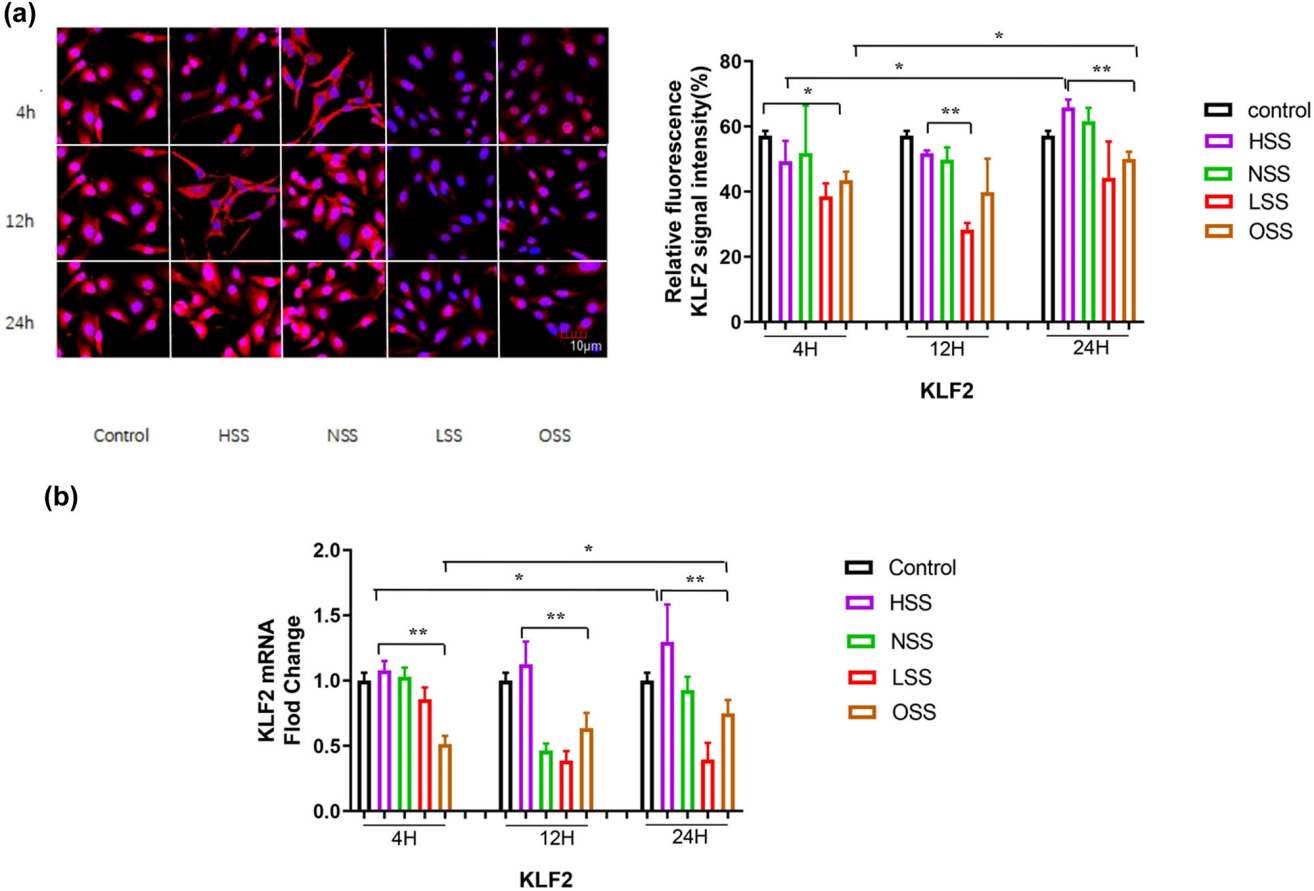
Immunofluorescence staining and RT-qPCR evaluating the effects of shear forces on the expression of KLF2. Red color represents KLF2 and blue color denotes the controls. (a) Immunofluorescence staining detecting the influence of different shearing forces on the expression of KLF2. The pictures are all enlarged to 600×. (b) RT-PCR assessing the effect of shearing force on the expression of KLF2 mRNA. HSS: high-strength laminar shear force. NSS: normal shear stress. LSS: low-strength laminar shear force. OSS: oscillating shear force. Control: physiological environment. Three independent experiments were done in this study, and triplicates were performed for each group.

The expression level and distribution pattern of KLF2 in HUVEC were detected by immunofluorescence staining and RT-qPCR (Figure 2). According to immunofluorescence detection, KLF2 was mainly expressed in the nucleus. With the effect of shearing force, the expression of KLF2 also increased in the cytoplasm (*p* < 0.05; Figure 2a). RT-qPCR results showed that, compared with the physiological state, the expression level of KLF2 was up-regulated under the action of physiological intensity and high-intensity laminar shear stress. The expression level of KLF2 was gradually up-regulated with the extension of the action time (*p <* 0.05) and reached the highest at 24 h (*p <* 0.01). Furthermore, the expression of KLF2 under HSS was higher than that under NSS (*p* < 0.05) (Figure 2b).

### 3.2 The effect of shear stress on the expression of Cav-1 in HUVEC under physiological conditions

**Figure 3:**
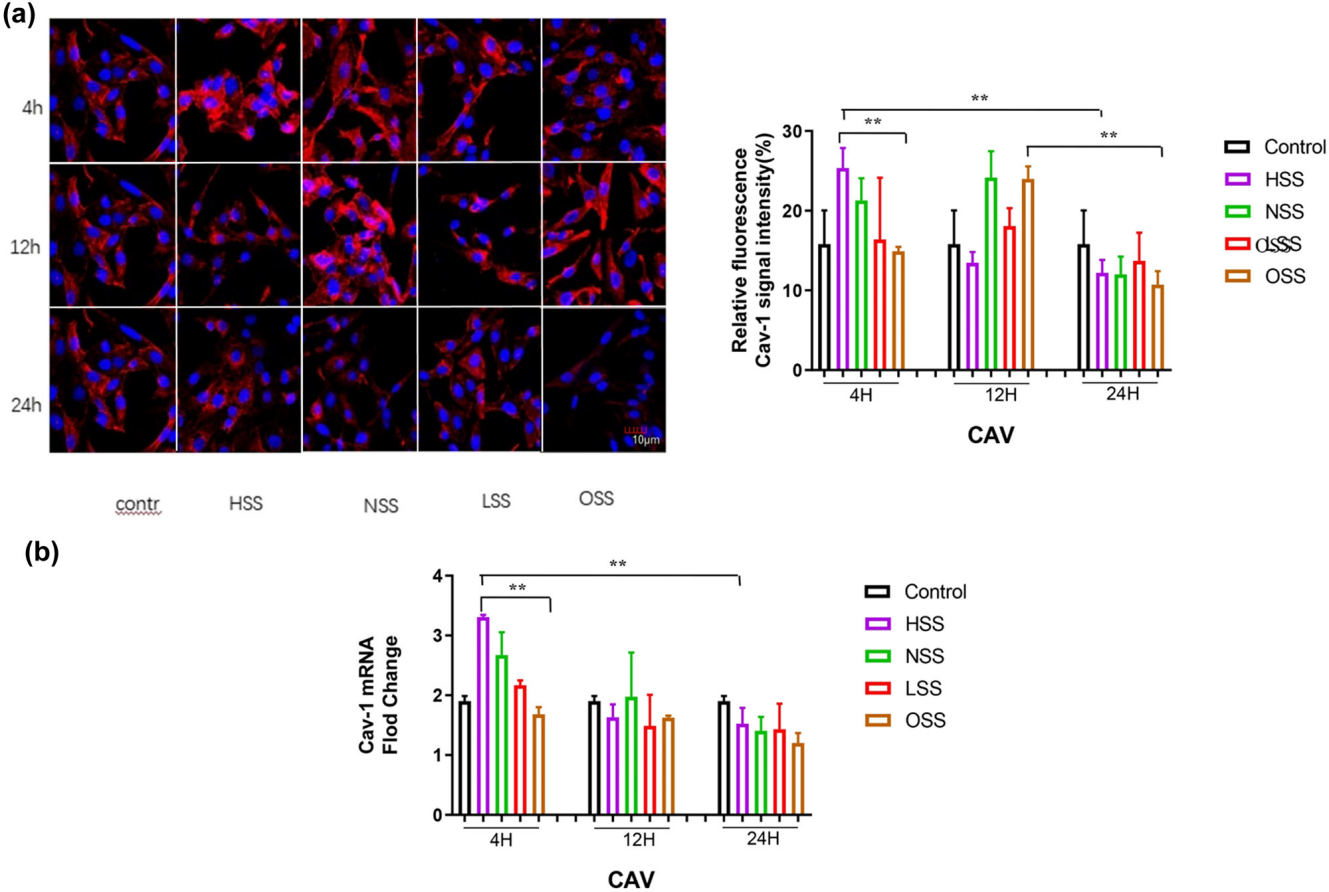
Immunofluorescence staining and RT-qPCR assessing the effect of shear forces on the expression of Cav-1. Red color represents Cav-1 and blue color denotes the controls. (a) Immunofluorescence staining detecting the effect of different shearing forces on Cav-1 expression. The pictures are all enlarged to 600×. (b) RT-PCR evaluating the effect of shearing force on Cav-1 mRNA expression. HSS: high-strength laminar shear force. NSS: normal shear stress. LSS: low-strength laminar shear force. OSS: oscillating shear force. Control: physiological environment. Three independent experiments were done in this study, and triplicates were performed for each group.

The expression level and distribution pattern of Cav-1 in HUVEC were detected by immunofluorescence staining and RT-qPCR. As illustrated in Figure 3a, immunofluorescence staining revealed that Cav-1 protein was mainly uniformly expressed in the cell membrane and cytoplasm. The results of RT-qPCR demonstrated that, compared with the physiological state, the mRNA expression level of Cav-1 was up-regulated under the action of physiological intensity and HSS, which was significantly higher than that of LSS and OSS (*p* < 0.05). With the extension of the action time, the expression level of Cav-1 gradually down-regulated, and it was the lowest at 24 h (*p* < 0.01), and the decrease became more prominent when the HSS was applied. The Cav-1 expression level was the lowest under OSS among the five groups (*p* < 0.01) (Figure 3b).

### 3.3 Effect of shear stress on the expression of eNOS in HUVEC under physiological conditions

**Figure 4:**
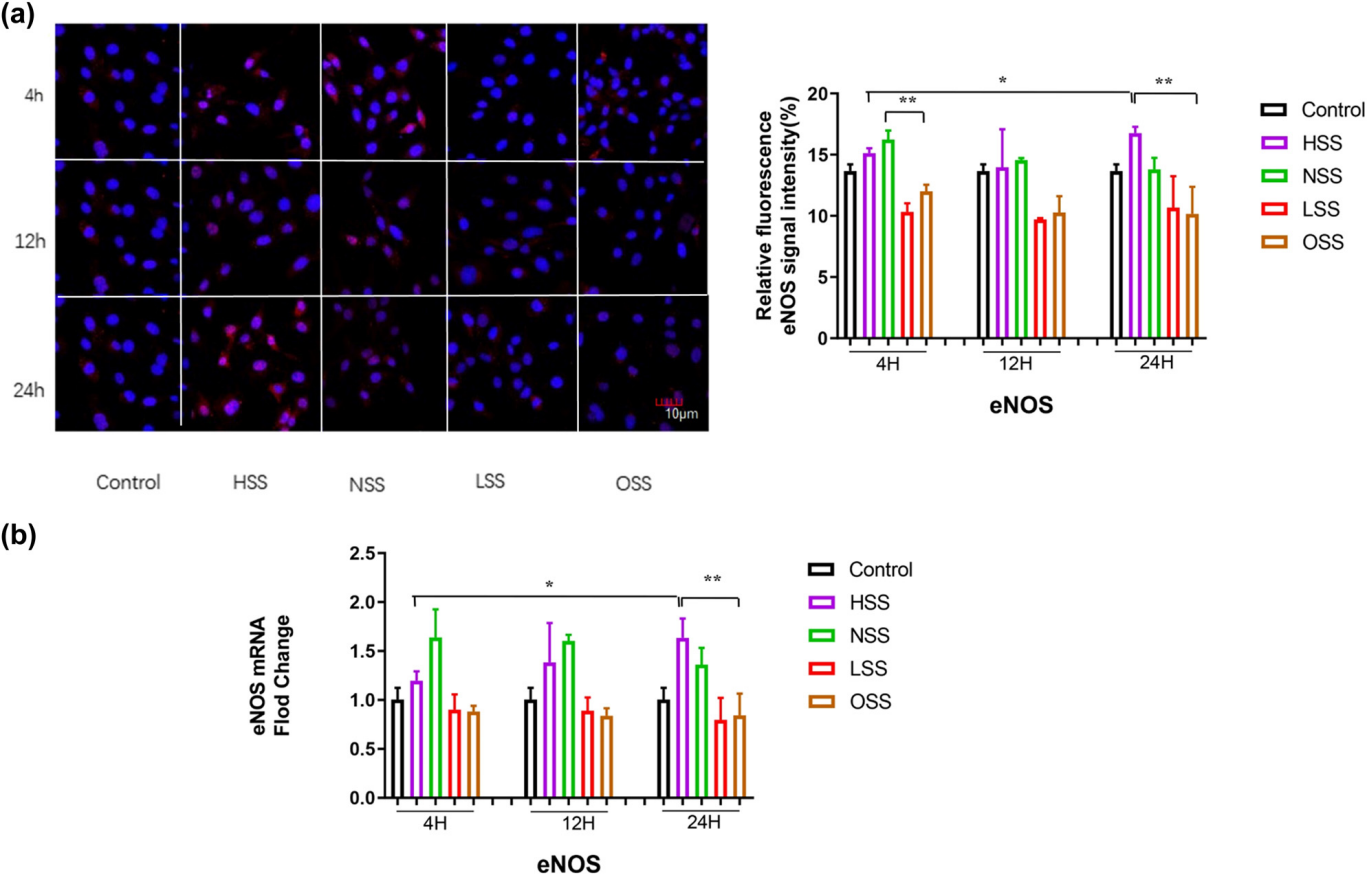
Immunofluorescence staining and RT-PCR assessing the effects of different shearing forces on the expression of eNOS. Red color represents eNOS and blue color denotes the controls. (a) Immunofluorescence detecting the effect of different shearing forces on eNOS expression. The pictures are all enlarged to 600×. (b) RT-PCR evaluating the effect of shearing force on eNOS mRNA expression. HSS: high-strength laminar shear force. NSS: normal shear stress. LSS: low-strength laminar shear force. OSS: oscillating shear force. Control: physiological environment. Three independent experiments were done in this study, and triplicates were performed for each group.

The protein and mRNA expression levels of eNOS in HUVEC were detected by immunofluorescence staining and RT-qPCR. Immunofluorescence staining showed that eNOS was mainly expressed in a granular form in the nucleus (Figure 4a). RT-qPCR indicated that, compared with the physiological state, the mRNA expression level of eNOS was up-regulated under the action of physiological intensity and HSS, which was significantly higher than that of LSS and OSS (*p* < 0.05). Under the action of HSS, the expression level of eNOS gradually increased and reached its highest at 24 h, which was significantly higher than that at 4 and 12 h (both *p* < 0.05). The expression level of eNOS under the action of HSS and OSS was significantly decreased (*p* < 0.05), as illustrated in Figure 4b.

### 3.4 The effect of shear stress on the expression of p-ERK in HUVEC under physiological conditions

**Figure 5:**
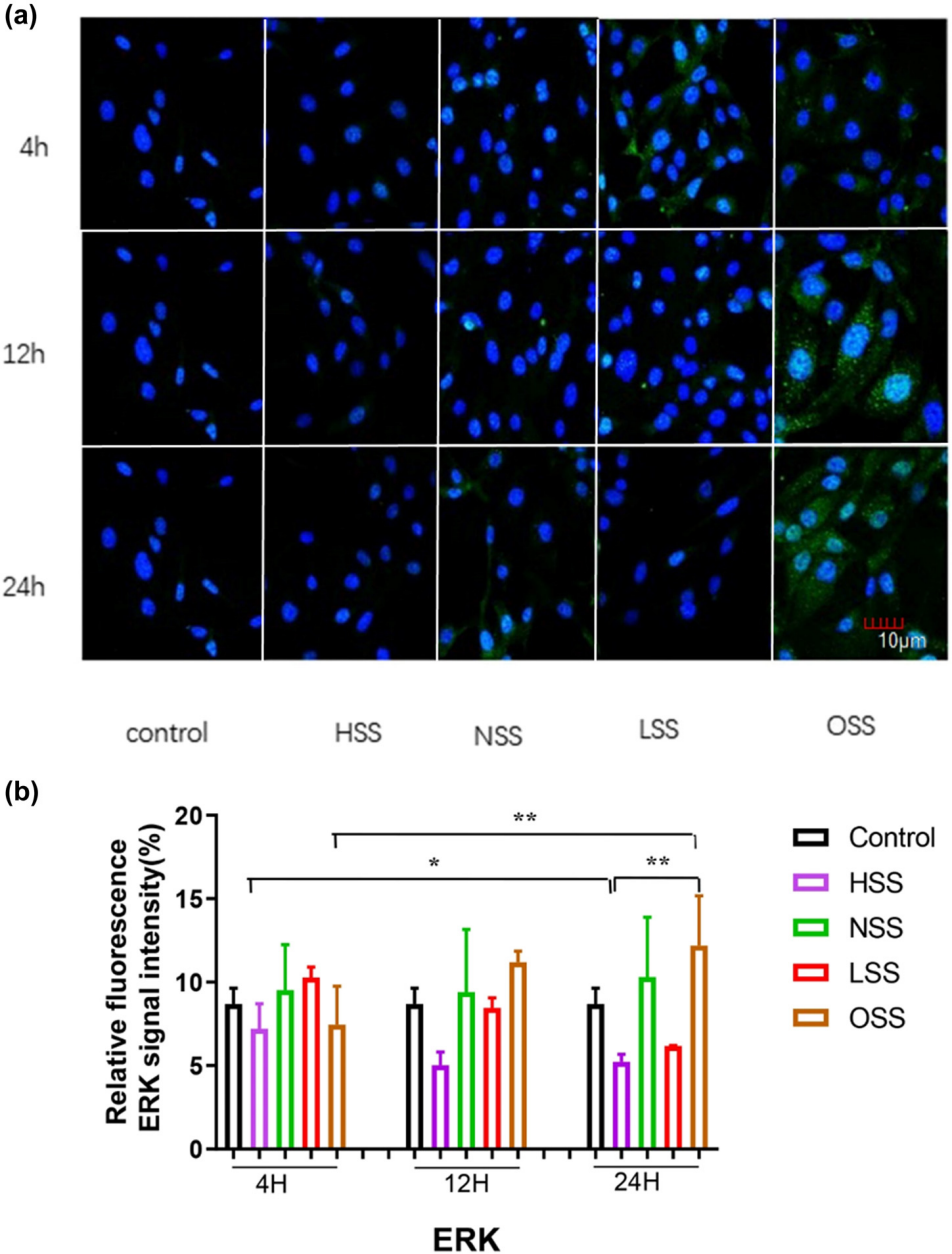
Immunofluorescence staining evaluating the effect of different shearing forces on ERK expression. Green color represents p-ERK and blue color denotes the controls. The picture shows the immunofluorescence detection of the effect of different shearing forces on p-ERK expression. The pictures are all enlarged to 600×. (a) Immunofluorescence detection of the effect of different shearing forces on p-ERK expression. (b) Evaluation of the effect of shearing force on p-ERK expression HSS: high-intensity laminar shear force. NSS: normal shear stress. LSS: low-intensity laminar shear force. OSS: oscillating shear force. Control: physiological environment. Three independent experiments were done in this study, and triplicates were performed for each group.

The expression level and distribution pattern of p-ERK in HUVEC were detected by immunofluorescence staining. Regarding the distribution pattern, immunofluorescence staining revealed that p-ERK protein was mainly expressed unevenly in the nucleus (Figure 5a). RT-qPCR showed that, compared with the physiological state, the expression level of p-ERK was significantly down-regulated under the action of high-intensity LSS, LSS, and OSS (*p* < 0.05). With the extension of the action time, the expression of p-ERK under the action of HSS was slightly down-regulated without a significant difference (*p* > 0.05). Under the action of physiological shear stress, the expression of p-ERK was significantly higher than that of the high-shear stress group, but it did not change significantly over time (*p* > 0.05). Under the action of LSS, the expression of p-ERK decreased with time. The expression of p-ERK under the action of OSS was lower than that under the physiological state (*p* < 0.05), and gradually up-regulated with the extension of time, and the expression intensity was the highest at 24 h (*p* < 0.01), as shown in Figure 5b.

### 3.5 The effect of Cav-1 on the expression of KLF2, eNOS, and p-ERK under physiological conditions

**Figure 6:**
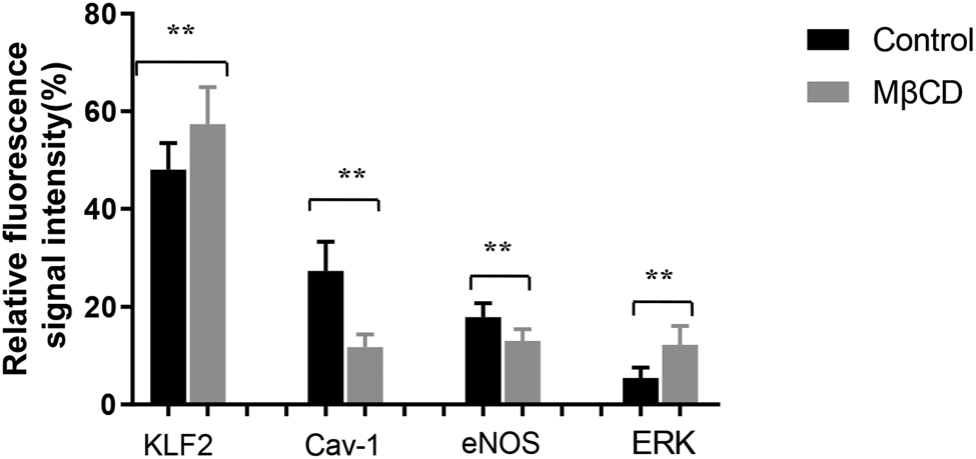
Effect of Cav-1 disruptor on the expression of KLF2/eNOS/ERK under oscillating shear force detected by immunofluorescence staining. Three independent experiments were done in this study, and triplicates were performed for each group.

Cav-1 structure was destroyed by agent MβCD at a concentration of 20 mM in the cultured ECs for 12 h, and gave a shock shear stress of 0 ± 4 dyn/cm^2^ for 24 h. Immunofluorescence staining was used to detect the expressions of Cav-1, KLF2, eNOS, and p-ERK in HUVEC. Cav-1 in the MβCD group was lower than that in the control group, indicating the block of Cav-1 expression. It was also showed that at 24 h after the same shock shear was applied, the relative protein expression levels of KLF2, eNOS, and p-ERK in the MβCD group were higher compared with those in the control group, as illustrated in Figure 6.

## 4 Discussion

The effect of shear stress on ECs is direct and continuous, affecting the function and structure of ECs. The migration of ECs plays an important role in various physiological processes such as blood vessel damage and repair and angiogenesis. The migration of blood flow, cytoskeleton, extracellular matrix, and other inhibitory factors are affected [15,16]. Shear stress can directly regulate a variety of cytokines in ECs. When ECs are subjected to a shearing force, these genes will be regulated through signal transduction pathways to regulate EC function [17]. In this study, we found that shear stress, especially OSS, may affect the function of ECs by directly acting on the Cav-1-mediated KLF2/eNOS/ERK signaling pathway.

KLF2 plays an important role in anti-inflammatory, anti-thrombotic, angiogenesis, and endocrine functions [18]. Studies have found that KLF2 is a key transcription factor leading to vascular inflammation. In KLF2-deleted T cells, there is overexpression of inflammatory factors (such as IFN-γ, IL-4, and TNF-α), indicating that KLF2 can inhibit the expression of the above inflammatory factors [19]. Blood flow shear stress is the only factor that induces the expression of KLF2 in ECs under physiological conditions [20]. In animal experiments, the expression of KLF2 on the outside of the celiac artery was significantly reduced, which is consistent with the results of *in vitro* experiments [21]. Experiments have found that LSS can regulate the expression of KLF2 through the adenylate-activated protein kinase (AMPK) signaling pathway [22]. Laminar shear stress can regulate nucleoli and transcriptional regulation of KLF2 expression. OSS can also inhibit the synthesis and expression of KLF2 by regulating the expression of thioredoxin binding protein. The combination of P53 and the specific inhibitory sequence on the KLF2 promoter inhibits the expression of KLF2, promotes coagulation, apoptosis, and affects vasodilation, resulting in EC dysfunction [23]. In addition, studies on statin lipid-lowering drugs and resveratrol have confirmed that they can up-regulate the expression of KLF2, play anti-coagulation, anti-atherosclerosis, and other effects, and protect the function of vascular ECs. Under the action of oscillating shear stress, KLF2 expression is inhibited, inflammatory factors are overexpressed, and EC function is impaired. In these inflammatory factors, it is also found that TNFα and IL-1β can inhibit the expression of KLF2 in ECs, and further aggravate the inflammatory response [24].

Cav-1 is a surface marker protein of EC cavern structure, which is obviously regulated by shear stress, and plays an important role in signal transduction [25].

The caveolae is a mechanoreceptor, which can sensitively feel the change of vascular shear stress [26]. When the shear stress increases within the physiological range, the caveolae structure will increase significantly, nitric oxide (NO) release will increase, and blood vessels will expand [27]. There is no change in the number of Cav-1, but Cav-1 will gather up the edge of ECs under the action of fluid and adjust the activation of some signal pathways. If the shear stress is significantly reduced or OSS occurs, the expression of Cav-1 will be weakened, and under the action of HSS, the expression of Cav-1 will increase. In addition, the increase in tension will cause the Cav-1-related outer structure to separate from the membrane, the pits will be flattened, and the corresponding protein regulation and signal transduction functions will change [28]. In this study, the increase of shear stress under physiological strength increased the expression of Cav-1, while the expression of Cav-1 was inhibited under OSS. Combined with previous experimental results, it shows that Cav-1 may be involved in the signal transduction pathway in ECs induced by shear stress, and it plays an important role in the mechanical conduction process of hemodynamics.

NO, as a second messenger molecule with high free radical activity, plays an important role in the maintenance of vascular tension and the stable regulation of blood pressure [29]. Among the subtypes of NOS, eNOS has biological activity in its dimer structure and can be expressed in specific cells (vascular ECs), and plays a vital role in the regulation of vascular wall function through regulating vascular tension and blood flow distribution, inhibiting vascular smooth muscle cell proliferation and platelet adhesion, preventing thrombosis, promoting angiogenesis, and participating in vascular remodeling and vascularization damage repair [30]. The study found that in the mouse model of knocking out the eNOS gene, the endothelial dysfunction of the whole body of the mouse was found, the vasodilation effect was significantly weakened, and severe hypertension appeared [31]. The migration of ECs involves a large number of proteins and cytokines such as NO and FAK. Previous studies have found that hemodynamic changes can affect the balance of NO and oxygen free radicals, and high intensity and directed shear stress can play a beneficial role in re-endothelialization and inflammation, and also affect vascular external remodeling [32].

The expression of KLF2 can directly regulate the expression of eNOS, and the extracellular signal-regulated kinase (ERK) signaling pathway can also regulate the expression of eNOS. In a turbulent state, by regulating Cav-1 in caveolin, the membrane-bound eNOS cycle is inhibited. In this study, the expression of eNOS was significantly up-regulated under the action of high-intensity and physiological-intensity LSS with high intensity and physiological intensity, while it was inhibited under low-intensity shear stress and OSS. MAPKs pathway exist in cells and can be activated by shear stress. Among the three pathways of MAPKs, ERK is the most important signal transduction pathway and a common pathway for a variety of growth factors to regulate cell growth, proliferation, and differentiation [33]. Under LSS, the ERK pathway inhibits eNOS activity, reduces NO release, and participates in the proliferation and migration of smooth muscle cells, which ultimately leads to vascular damage and intimal hyperplasia [34]. After treatment with the caveolae structure-disrupting agent, the number of Cav-1 decreased, the intracellular caveolin increased, and ERK activity (p-ERK) was found to increase. When the endogenous caveolin is down-regulated, late ERK activity completely abates. The mechanical force acting on Cav causes the ROS system, MAPK-ERK system, NO system, and NF-кB system to convert signals into biological signals that act on cells and nuclei, leading to immediate and long-term vascular response, causing EC to divide and proliferate smooth muscle migration. In this study, the expression of Cav-1 was reduced and the expression of eNOS was weakened under the action of the Cav-1 structure disruptor, indicating that the expression of eNOS can be inhibited by reducing Cav-1.

## 5 Conclusions

Under physiological conditions and the action of high-strength and physiological-strength LSS, the expression of KLF2, Cav-1, and eNOS increases, and the expression of p-ERK decreases. Under physiological conditions, the expression levels of KLF2, Cav-1, and eNOS are reduced, and the expression of p-ERK is increased under the action of oscillating shear stress and low-intensity laminar shear stress. After the action of the Cav-1 structure destroyer, the expression of eNOS is weakened, and the expression of KLF2 and p-ERK is increased under the action of OSS. It is indicated that OSS may directly affect the function of ECs by directly acting on Cav-1-mediated KLF2/eNOS/ERK signaling pathway. These initial findings may result in the identification of novel therapeutic targets to improve ECs function.
